# The Effect of Landscape Environmental Factors on Gene Flow of Red Deer (*Cervus canadensis xanthopygus*) in the Southern of the Greater Khingan Mountains, China

**DOI:** 10.3390/biology12040576

**Published:** 2023-04-10

**Authors:** Zheng Li, Jinhao Guo, Yang Hong, Ning Zhang, Minghai Zhang

**Affiliations:** College of Wildlife and Protected Area, Northeast Forestry University, Harbin 150040, China

**Keywords:** red deer, genetic diversity, genetic differentiation, gene flow, landscape environmental variables, dispersal

## Abstract

**Simple Summary:**

Using molecular techniques, we assessed the current situation of genetic structure and gene flow among different red deer groups living in the southern part of the Greater Khingan Mountains, the main distribution area of red deer in China. The result showed that the overall genetic diversity of red deer in the study area was intermediate. Significant genetic differentiation was observed, and a significant correlation was found between landscape variables and genetic differentiation, especially roads, altitude, and settlements, which were considered the main factors affecting the gene flow of red deer in this region. The research suggested that we should pay more attention to artificial landscapes and the supervision of human activities, and the distribution of human-made landscapes should avoid crossing the main habitats of red deer as much as possible.

**Abstract:**

Red deer (*Cervus canadensis xanthopygus*) living in the north of China are restricted and threatened due to human activities and the changes in the natural environment, which influence the dispersal and effective gene flow between different groups of red deer. Effective gene flow plays an important role in maintaining genetic diversity and structure and ensuring population health. In order to evaluate the genetic diversity level and understand the gene flow between different red deer groups, 231 fresh fecal samples were collected from the southern part of the Greater Khingan Mountains, China. A microsatellite marker was used for genetic analysis. The results showed that the genetic diversity of red deer was intermediate in this region. Significant genetic differentiation among different groups was found in the main distribution area (*p* < 0.01) using *F*-statistics and the program STRUCTURE. Different degrees of gene flow existed in red deer groups, and the roads (*importance* = 40.9), elevation (*importance* = 38.6), and settlements (*importance* = 14.1) exerted main effects on gene flow between red deer groups. Human-made factors should be noticed and strictly supervised in this region to avoid excessive disturbance to the normal movement of the red deer. Further conservation and management of red deer should reduce the intensity of vehicular traffic in the concentrated distribution areas of red deer, especially during the heat season. This research helps us better understand the genetic level and health status of red deer in the southern part of the Greater Khingan Mountains and provides theoretical references for protecting and restoring the red deer populations in China.

## 1. Introduction

Dispersal is a central process in ecology and evolution [1,2,3]. In nature, the effective gene flow between individuals or populations is an important manifestation of wildlife migration or diffusion, which could lead to genetic diversity and structure changes based on the population level [4]. Maintaining sufficient effective gene flow plays an important role in ensuring population health and enhancing adaptability to the environment [5]. However, gene flow is affected by various factors, including food, natural enemies, reproductive ability, etc. In particular, it is mainly affected by habitat connectivity [6]. Habitat connectivity is always related to environmental factors and landscape features. As early as 1947, Fisher et al. proposed that the distribution pattern of landscapes affected the dispersal of wildlife and gene flow [7]. The effects of landscape features on gene flow are both positive and negative, which could inhibit or facilitate wildlife dispersal and gene exchange [8,9,10,11,12,13,14]. The red deer (*Cervus canadensis xanthopygus*) is an essential forest mammal in northern China, which plays an important role in maintaining the stability of forest ecosystems and the number of large carnivores, such as the Amur tiger [15]. However, the number of red deer declined rapidly in China due to hunting and logging before the 19th century [16]. At present, although considerable efforts have been made to protect the red deer population, its recovery rate is slow. The effects of human activities and changes in natural resources on red deer still need attention [17,18]. The assessment of the genetic status of red deer and the analysis of the factors affecting gene flow can effectively help us understand the health status of the red deer population, avoid potential threats and risks, and provide a theoretical basis and scientific references for timely updating and modifying management and protection measures.

The Greater Khingan Mountains, located in northeast China, have abundant wildlife resources. The Gaogesitai region is located in the southern part of the Greater Khingan Mountains, currently known as the most important distribution area of red deer in China [19]. However, the current genetic level and health status of the red deer population living in this region are still unknown. In addition, there were settlements and a well-developed transportation system, mainly used for transporting wood at the end of the 20th century. After establishing the nature reserve in 2011, these roads were used for vehicle traffic and daily patrol work of the natural reserve. There were still settlements in the main distribution areas of red deer [20]. The vehicles and human activities might affect the dispersal and gene flow of red deer. Therefore, it is necessary to evaluate the influence of environmental variables, including human factors, on the effective gene flow between red deer communities in this region, which is important for assessing the health of red deer populations in this region and ensuring the stability of red deer populations in China.

In this study, therefore, we investigated the genetic diversity of red deer in the southern part of the Greater Khingan Mountains to understand the impact of landscape features on red deer from a genetic perspective. The main objectives of the study are as follows: (1) evaluating the current level of genetic diversity; (2) revealing if potential genetic differentiation existed between different groups of red deer; and (3) analyzing the effects of environmental factors on gene flow.

## 2. Materials and Methods

### 2.1. Study Area

**Figure 1:**
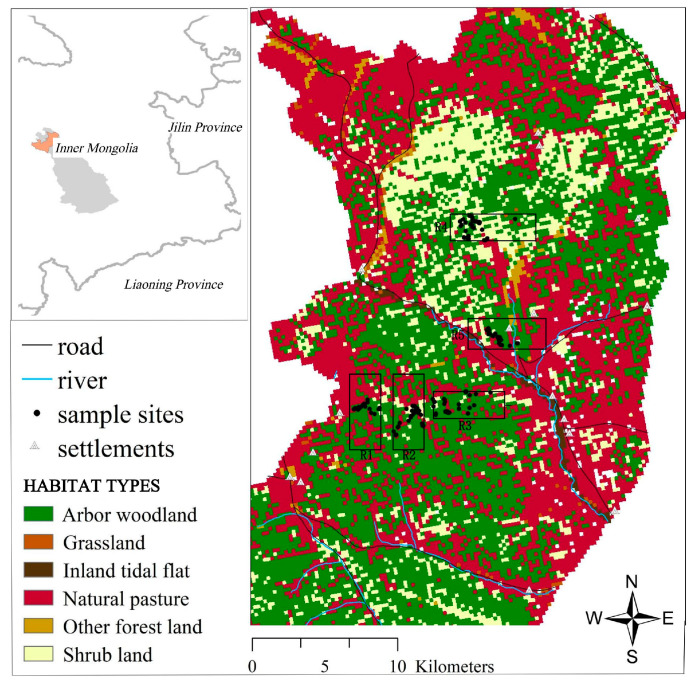
Map of the study area. The points in the figure represent the sampling sites, and R1, R2, R3, R4, and R5 represent five different red deer groups. R1, Xishabutai (*n* = 29); R2, Sitehehundi (*n* = 57); R3, Shabutai (*n* = 55); R4, Luchangxi (*n* = 61); R5, Changbu (*n* = 29).

Gaogesitai region is located in the southern part of the Greater Khingan Mountains, belonging to Chifeng City, Inner Mongolia, China, with a total area of 1062.84 km^2^ (119°03′30″–119°39’08″ E, 44°41′03″–45°08′44″ N, Figure 1). This region is an important distribution area of red deer, with intact habitat and abundant red deer resources (average density = 1.11/km^2^) [18]. The region has forests, shrubs, grasslands, and wetlands ecosystems, with an altitude of 700–1600 m and a slope of 0–54°. The rich vegetation and water resources provide suitable habitats for red deer to survive and reproduce. In winter, the average snow cover is 30 d, and the longest snow cover is 100 d. The coldest month is January, with an average temperature of −16 °C and an extremely low temperature of −42 °C. In addition to the existing natural landscapes, there are artificial landscapes, including settlements and roads. Settlements include herders, staff, and workers. Roads are mainly used for traffic and daily patrol. Based on previous studies, local landscape environmental factors are likely to affect the dispersal and gene flow between different groups of red deer [21,22].

### 2.2. Sample Collection

During the winter of 2020, based on the results of previous infrared camera photography and the experience of local forest rangers, we conducted field surveys in five areas with high activity of the red deer in R1, R2, R3, R4, and R5 (full names and sample sizes are given in the legend of Figure 1). In January 2021, fresh fecal samples (<24 h) were collected by tracking the snowy trail chains of red deer. Disposable sterile collection bags were used for sampling, and the samples were immediately stored in liquid nitrogen. After sampling, the samples were shipped to the laboratory and stored in an ultra-low-temperature refrigerator at −80 °C for preservation. Finally, 231 samples were collected for this study.

### 2.3. DNA Extraction and Species Identification

We strictly applied the laboratory protocols throughout the experiment to prevent contamination by alien DNA and PCR products. Fecal DNA extraction was performed using the QIAamp Fast DNA Stool Mini Kit (Qiagen, Hilden, Germany) according to the manufacturer’s instructions. Using mitochondrial cytochrome *b* amplification primer L14724: 5′-CGA GAT CTG AAA AAC CAT CGT TG-3′; H15149: 5′-AAA CTG CAG CCC CTC AGA ATG ATA TTT GTC CTC A-3′ [23,24] for PCR amplification of fecal DNA (400–500 bp). The amplification system was shown in Table S1. Reaction conditions: pre-denaturation at 95 °C for 3 min; denaturation at 95 °C for 30 s; annealing at 53 °C for 30 s; extension at 72 °C for 30 s, for 35 cycles; then, extension at 72 °C for 5 min; and storage at 4 °C. Muscle DNA stored in the laboratory was used as a positive control for each amplification (from naturally deceased females found in the field). A negative control without DNA was added along with the amplification to monitor contamination. Each DNA extraction was amplified and indexed in three independent PCR reactions. These PCR replicates were used as technical replicates to remove the false-positive results [25]. The PCR products were examined on 1.5% agarose gel electrophoresis. The positive products were sent to the Shanghai Shengong Biological Company for purification and two-way sequencing. The forward and reverse sequences were spliced, aligned, and corrected by DNAStar software. Red deer species were identified by comparison with the NCBI GenBank reference databases [26]. If a sample was not identified as red deer, it was discarded from further analysis. Cervidae in the study area all have publicly available DNA references for the mitochondrial markers. Therefore, operational taxonomic units (OTUs) can be classified at the level of taxonomic species.

### 2.4. Individual Identification

Based on published studies on red deer, eight pairs of microsatellite primers (ETH225, T501, T156, BM848, T530, DM45, N, T507) were used for individual identification [27,28,29]. The 5′ end of the upstream primer in each microsatellite locus was fluorescently labeled (Table S2). The reaction conditions were the same as those used for species identification. 

The multiple-tube approach was used to carry out several polymerase chain reactions at each locus for reliable genotypes [30]. The RelioType software was used to accept genotypes that achieved a 95% estimated probability of reliability [31]. Each locus was amplified by polymerase chain reaction at least four times. The PCR products were examined on 2% agarose gel electrophoresis. Genotypes were determined using an ABI 3730XL sequencer and the GeneMapper software package (Applied Biosystems Inc., Waltham, MA, USA). The software Excel microsatellite tool kit was used to look for matching genotypes in the data [32]. The principles for judging that different samples belong to the same individual are as follows: (1) the genotypes are the same at all loci, and (2) only one allele at one locus varies [33]. To assess genotype quality, Gimlet version 1.3.3 was used to construct consensus genotypes and estimate the false allele (FA) and allele dropout (ADO) rates [34].

### 2.5. Genetic Diversity Analysis

For microsatellite data, GenAlEx version 6.5 was used to transform the allele data and calculate the number of alleles (*N*_a_), the effective allele number (*N*_e_), the expected heterozygosity (*H*_o_), and the expected heterozygosity (*He*) [35]. The polymorphism information content (*PIC*) of microsatellite loci was calculated by Cervus version 3.0. Further, the individual identification probability *P*_ID_ of eight microsatellite loci was evaluated by Gimlet version 1.3.3 [34]. The Microchecker version 2.2.0.3 was used to detect null alleles at microsatellite loci [36]. Genepop version 4.0 was used to measure whether the population and each locus deviated from Hardy–Weinberg equilibrium (HWE) [37], and linkage disequilibrium (LD) between each locus was also examined. For LD and HWE tests, *p*-values were generated by the Markov chain method, and significance was corrected by the Bonferroni method [38].

### 2.6. Genetic Differentiation Analysis

Two methods were used to analyze the population differentiation. First, Genepop version 4.0 was used to calculate the genetic differentiation index (*F*_st_) between the pairs of sampling sites [39]. Fisher’s exact test was used for significance values, and the Markov chain (MCMC) was set as 10,000 dememorization steps, 100 batches, and 500 iterations per batch [40,41].

The prior subdivision of the population by *F*-statistic can be subjective, leading to some bias in the estimates of population structure [42]. Therefore, a Bayesian clustering method was implemented in the program STRUCTURE version 2.3 to reduce the error when defining the population to analyze the genetic structure [43]. The software inferred the number of potential genetic clusters (*K*) using an individual-based Bayesian clustering method without defining populations a priori. The most likely number of populations (*K*) was estimated by conducting 20 independent runs for *K* = 1–5, using a burn-in of 10^5^ replications and 50,000 Markov chain Monte Carlo steps, and assuming the admixture model with correlated allele frequencies. The running results were analyzed by Structure Harvester Web “http://taylor0.biology.ucla.edu/struct_harvest/ (accessed on 15 March 2023)” [44]. Then, the curves of Ln Pr (X|K) and delta *K* were calculated, and the best *K* value was determined by maximizing the delta *K*. CLUMPP version 1.1.2 [45] was used to average the results for the *K* value, and finally, plotted them with Distruct version 1.1 [46]. In addition, we further analyzed the genetic structure using the discriminant analysis of principal components (DAPC) with a non-Bayesian approach. DAPC was performed in R version 3.3.1 using the “adegenet” package to determine the clusters of groups according to the Bayesian Information Criterion (BIC) [47].

### 2.7. Isolation-by-Distance (IBD) Analysis

An Isolation-by-distance (IBD) test was performed in the study area to assess potential distance barriers to gene flow and investigate if genetic differentiation between sampling sites followed the IBD model [48,49]. The Center of Mass and Xtools Pro in ArcGIS version 10.3 were used to calculate the Euclidean distance between pairwise red deer groups. The pairwise genetic distance matrix was generated by *F*_st_/(1-*F*_st_). The natural logarithmic transformation of Euclidean distance generated the geographical distance matrix. The Mantel test [50] was performed using GenAlEx version 6.5 to test the correlation between genotype and geographical distance [51]. The *p*-value was obtained after 10,000 displacement tests.

### 2.8. Source of Environmental Variables

The environmental variables included vegetation, topography, water, and disturbance. Moreover, normalized difference vegetation index (NDVI) and habitat types were considered vegetation factors. The elevation and slope were considered topography factors. The tributaries in this area were considered water factors. Settlements and roads were considered disturbance factors. The vegetation factors were derived from MODIS images “https://modis.gsfc.nasa.gov (accessed on 20 December 2022)” and the Third National land resource survey (The survey is a national land and resources survey of China. According to the unified national standards, the survey uses remote sensing, surveying and mapping, geographic information, Internet, and other technologies to fully grasp the utilization of existing land resources in China, including the area and distribution of various habitats.). The topography factors were extracted from the Digital topographic Elevation Model (DEM) on the Geospatial Data Cloud platform of the Computer Network Information Center, Chinese Academy of Sciences “http://www.gscloud.cn/ (accessed on 20 December 2022)”. The water factors were based on the Euclidean distance layer generated from the river distribution layer in the study area, which can reflect the distance of sample sites from the nearest water source. The disturbance factors were the Euclidian distance layer generated by the human disturbance (settlements and roads) and the sample sites obtained from field surveys and stock maps in the study area. The details about environmental variables are given in Table 1.

### 2.9. The Relationship between Landscape Environmental Variables and Gene Flow

#### 2.9.1. The Dispersal Resistance of Study Area

The sample sites and environmental variables were input into MaxEnt model version 3.4.1. Before inputting the environment variables, river elements were removed because of their high autocorrelation with other variables. 75% of the red deer distribution points were selected as the training set to establish the prediction model, and the remaining 25% were used as the test set to verify the model. Other parameters selected the default value of the model. The knife-cutting method was selected in the environmental parameter settings, and the analysis results were output as an ASCII file. Furthermore, the habitat suitability index (HSI) between 0 (unsuitable) and 1 (most suitable) was evaluated. The MaxEnt model was considered an effective tool for habitat assessment [52].

This study used the area enclosed by the ROC and the abscissa to evaluate the model’s accuracy. The closer the AUC value was to 1, the better the model predicted [53]. The dispersal resistance was calculated by 1-HSI. The area with less suitable habitat had higher resistance to the dispersal of red deer [54,55,56].

#### 2.9.2. Assessment of Dispersal Probability and Correlation with Gene Flow

Effective gene exchange among red deer is caused by dispersal, movement, and interpenetration among individuals or populations of red deer. The suitable area for red deer has less movement resistance and greater dispersal possibility, which indicates more extensive potential gene flow; on the contrary, the unsuitable area for red deer has higher movement resistance and less dispersal possibility, which indicates smaller potential gene flow. In order to better understand the relationship between gene flow, dispersal, and environmental variables, the circuit model and the least-cost path model were performed to obtain possible dispersal routes and dispersal cost distance.

Each red deer group was used as the ecological source, defined as source N. Other groups were used as the target N, producing independent ecological sources and one-to-one corresponding target sources. Based on the habitat suitability layer generated by the MaxEnt model, the resistance layer generated by 1-HSI was used as the base data source. The base data source was input into the circuit model. Then, the probability layer of red deer dispersing through the landscapes was obtained using Circuitscape version 4.0 [57]. The model parameters were selected in the pair-model pattern. Finally, the sampling sites, resistance, and probability layers of red deer dispersing through the landscapes were input into the least-cost path model to obtain the least-cost paths between different red deer groups [58]. In order to analyze the relationship between potential dispersal and gene flow, the least-cost path distance matrix and the genetic distance matrix among red deer groups were established. The Mantel test was performed in GenAlEx version 6.5 to obtain the *p*-values with 10,000 permutation tests.

## 3. Results

### 3.1. Species Identification and Individual Identification

231 fecal samples were collected in the study area, and 212 DNA samples were successfully extracted, with a sample utilization rate of 91.77%. The Cyt *b* gene was successfully amplified from 212 samples (424 bp). After a Blast comparison, 199 samples were finally identified as red deer.

**Figure 2:**
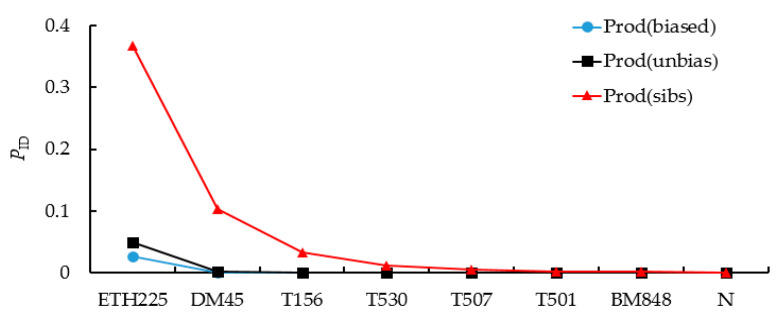
The probability of identity (*P*_ID_) curve generated eight microsatellite loci.

Gimlet analysis showed that the combined Prod(unbias) of the 8 microsatellite loci was 6.888 × 10^−10^. Even in the case of full sibs, the probability of misjudgment, Prod(sibs), was only 0.0546%. When the most polymorphic loci failed to amplify, the Prod(sibs) increased to 0.46%, which was still less than 1% (Figure 2). Therefore, 193 DNA samples were successfully genotyped at ≥7 microsatellite loci (96.98% amplification success rate) with 95% reliability. The result of individual identification showed that 172 independent individuals of red deer were identified from 193 red deer fecal samples, which were used for subsequent analysis (Table 2). Moreover, the false allele (FA) and allele dropout (ADO) rates were 0.02 and 0.01, respectively.

### 3.2. Genetic Diversity Analysis

The results of Microchecker showed no null alleles or allele loss, indicating that the genotyping results were reliable and could be used for subsequent analysis. The red deer in the study area significantly deviated from the Hardy–Weinberg equilibrium. However, the fixation index (*F*_is_) was negative (*F*_is_ = −0.051), which proved no effect of a null allele or inbreeding. There was no linkage disequilibrium among the eight loci. Genotyping data were therefore available for subsequent population genetic analysis.

The number of alleles (*N*_a_) and effective alleles (*N*_e_) were 8.0 ± 0.51 and 4.6 ± 0.32, respectively. A significant difference was found between *N*_a_ and *N*_e_ (*p* < 0.01). Allelic richness (*AR*) was 5.978. The polymorphic information content (*PIC*) was 0.724. The expected heterozygosity (*H*_e_) was 0.737 ± 0.018, and the observed heterozygosity (*H*_o_) was 0.767 ± 0.031. A significant difference was found between observed heterozygosity and expected heterozygosity (*p* < 0.05). The details are shown in Table 3.

### 3.3. Genetic Differentiation Analysis

The overall population differentiation was relatively low in this region (*F*_st_ = 0.014 < 0.05). The *F*_st_ among pairwise red deer groups ranged from 0.0041 to 0.0223. The details are shown in Table 4. A significant difference in genetic differentiation was found after Fisher’s exact test, which was R1-R2, R1-R3, R1-R4, R1-R5, R2-R3, R2-R4, R2-R5, and R3-R4 (*p* < 0.01).

**Figure 3:**
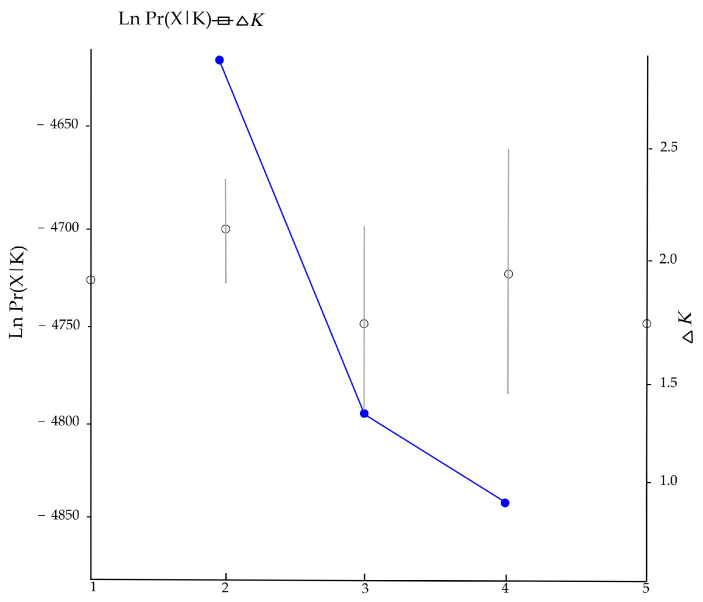
Changing trends of Ln Pr(X|K) and Delta *K* from STRUCTURE clustering results.

**Figure 4:**
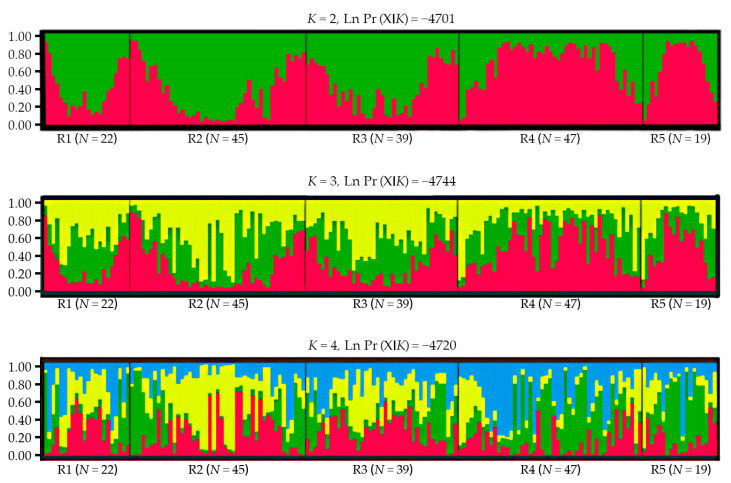
Bayesian clustering results of red deer for the microsatellite (*K* = 2–4). A line represents each individual (*N* = 172), different colors represent different groups, and the proportions of different colors in the lines are the probability that an individual was assigned to a certain group. Individual numbers of each sampling group are shown at the bottom of the figure.

**Figure 5:**
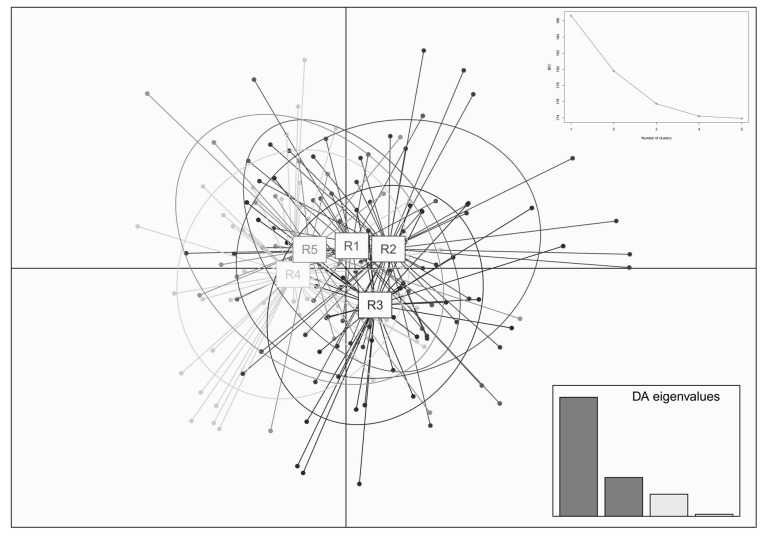
Genetic clusters with DAPC method for red deer based on microsatellite data. The figure included the cluster graph of individuals from each group and the broken line graph corresponding to the cluster number *K* and BIC values.

The Bayesian clustering showed that delta *K* reached its highest when *K* = 2, with the average Ln P(X|K) = −4701. When *K* > 2, Ln P(X|K) decreased significantly, and the variance between independent operations increased. Therefore, *K* = 2 might be the most probable number of clusters (Figure 3). Each individual at *K* = 2–4 was grouped and analyzed, and the grouping results of the 172 individuals are shown in Figure 4. When *K* = 2, the study area samples were divided into red and green clusters. Red clusters included most individuals living in the R4 and R5 groups, while the green group included the majority of individuals living in the R1, R2, and R3 groups. According to the multi-locus genotype assignment, the degree of genetic differentiation between R2 and R4 was the most obvious. The results of DAPC showed that all individuals were subdivided into five cluster groups, but the five clusters were not clearly separated, indicating that red deer communicated to different degrees between each cluster (Figure 5). And lower genetic differentiation and a more similar genetic composition could be found in R4 and R5, and R1, R2, and R3.

### 3.4. Isolation by Distance Analysis

**Figure 6:**
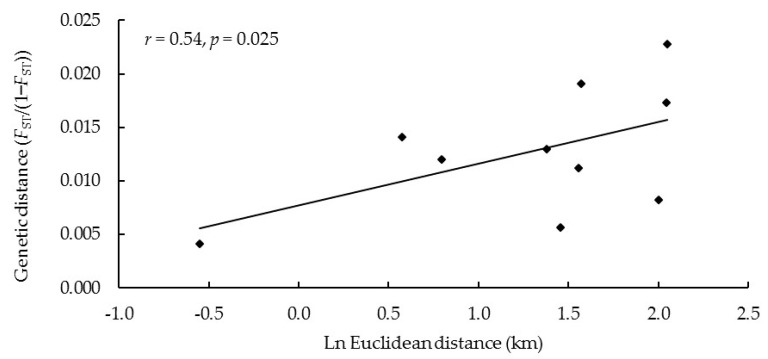
Mantel test of Euclidean distance and genetic distance between different red deer groups.

The average geographical distance among red deer groups was 4.5 km. Among them, the distance was the furthest between R2 and R4 and the closest between R1 and R2. The results of the Mantel test showed that there was a significant correlation between genetic differentiation and geographical distance (*r* = 0.54, *p* = 0.025 < 0.05) (Figure 6).

### 3.5. Relationship between Gene Flow and Environmental Variables

The Maxent model with 10 replicates showed that the AUC index of the dataset used in the research reached 0.976, which indicated a good evaluation and explanation of the results. Roads (*importance* = 40.9%), elevation (*importance* = 38.6%), and settlements (*importance* = 14.1%) were the main landscape features affecting the dispersal of red deer. The closer the distance to the road, the greater the dispersal resistance of red deer and the lower the dispersal possibility. At the altitude range of 750–900 m, the dispersal resistance decreases gradually and increases rapidly after 900 m. And within a certain range, the farther away from the settlements, the smaller the dispersal resistance (Figure S1). Habitat type (*importance* = 4.0%), NDVI (*importance* = 2.2%), and slope (*importance* = 0.2%) had less effect on the dispersal of red deer.

**Figure 7:**
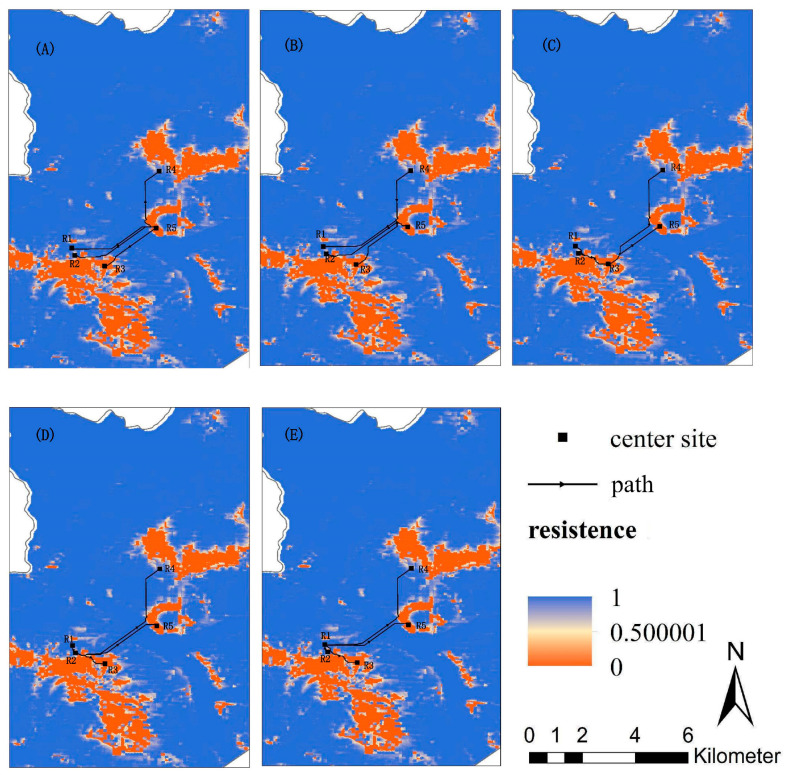
The least-cost paths among main distribution areas: (**A**) The least-cost path from distribution R5 to other distributions; (**B**) Distribution R4 to other distributions; (**C**) Distribution R3 to other distributions; (**D**) Distribution R2 to other distributions; (**E**) Distribution R1 to other distributions.

Based on the MaxEnt model, six types of landscape variables in the study area were assigned a dispersal cost value, and the least-cost paths were generated based on the circuit model and the least-cost path model. And the least-cost paths among red deer groups are shown in Figure 7.

**Figure 8:**
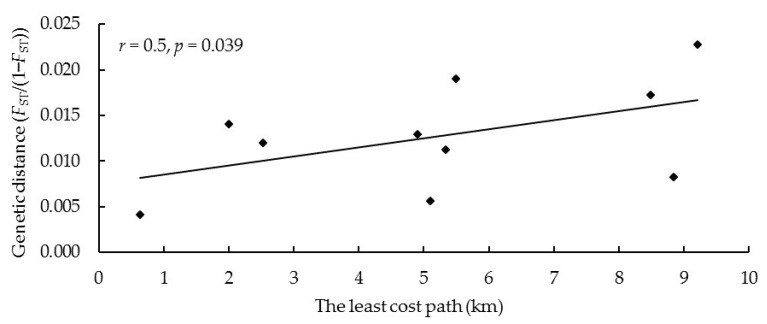
Mantel test of the least-cost path and genetic distance between five sampling sites based on the microsatellite data.

The results of the Mantel test showed a significantly positive correlation between the cost distance of dispersal and the genetic distance of red deer groups (*r* = 0.5, *p* = 0.039) (Figure 8).

## 4. Discussion

The studies focused on genetics and population dynamics could help us understand the health status of wildlife and avoid potential threats and risks. We investigated the genetic diversity level and structure of red deer living in the southern part of the Greater Khingan Mountains, China, which were considered dominant groups of red deer in China. The results suggested that the red deer in the southern part of the Greater Khingan Mountains might face the risk of decreasing their ability to adapt to the environment and forming local populations. We used microsatellite markers to understand the genetic status of red deer, as the microsatellites are suitable for inferring recent population genetic events [59]. In this study, eight microsatellite loci were used for analysis. There was a significant difference between the number of alleles and effective alleles (*p* < 0.01), which might lead to a risk of allele loss in the future. Heterozygosity is one of the most effective methods to quantify the level of genetic diversity. Gao (2020) studied the Alashan red deer (*Cervus elaphus alashanicus*) distributed in the Helan Mountains, Ningxia, and Inner Mongolia, China, and the results showed that the observed heterozygosity in the wild population was high (*H*_o_ = 0.792) [60]. Zhou (2015) conducted a population study on the Tianshan red deer (*Cervus elaphus songaricus*) distributed in the Tianshan Mountains, Xinjiang, China, and found that the overall genetic diversity was high (*H*_o_ = 0.850, *H*_e_ = 0.710) [61]. Based on 172 red deer individuals in this study, the observed heterozygosity (*H*_o_) was 0.767 and the expected heterozygosity (*H*_e_) was 0.737, which were lower than those of the Alashan red deer and Tianshan red deer. Compared with the results of other red deer subspecies based on microsatellite markers, the genetic diversity of red deer in the southern Greater Khingan Mountains in this study was at a medium level (Table 5).

The interference of human activities and the change in nature may lead to the hindrance of gene exchange, the decrease in genetic diversity, and the increase in genetic differentiation, which is easy to form endangered populations [66]. We found a certain degree of genetic differentiation in the study area (*F*_st_ = 0.014). The highest *F*_st_ was between R2 and R4, and the lowest *F*_st_ was between R1 and R2, indicating relatively strong effective gene flow between R1 and R2 and relatively low effective gene flow between R2 and R4. We found a positive correlation between genetic and geographical distance (*p* < 0.05). The longer geographical distance inhibited the dispersal of red deer, reduced gene flow between different red deer groups, and led to increased genetic differentiation [48,67,68].

However, the landscape and environmental variables might be important factors affecting the dispersal of red deer, which must be considered when studying gene flow among red deer groups [69,70]. This research found a significant correlation between genetic distance and cumulative dispersal resistance of landscape environmental variables (*p* < 0.05). Roads (40.9%), altitude (38.6%), and settlements (14.1%) were the main factors affecting the gene flow between red deer groups. The habitat types, NDVI, and slope had little influence on the gene flow of red deer, with an importance index of 4.0%, 2.2%, and 0.2%, respectively. According to the distribution of red deer, there were roads and settlements between R2 and R4. According to the relationship between dispersal resistance and distance to roads and settlements, the closer the distance to roads and settlements, the greater the diffusion resistance (Figure S1). So the red deer close to roads and settlements have higher dispersal costs. These environmental variables reduced effective gene flow, especially in linear landscapes, which affected the gene flow patterns at multiple spatial scales and further affected genetic differentiation [71,72,73]. Near the roads and settlements, frequent human activities and land use changes affected the dispersal pattern. Some other studies on red deer also confirmed that roads and settlements were the main limiting factors for the distribution and dispersal of red deer [74,75]. The results of STRUCTURE also demonstrated this, which suggested that all individuals of red deer could be divided into two clusters, and the R1, R2, and R3 groups of red deer on the southern side of roads belonged to one cluster, and R4 and R5 on the northern side of roads belonged to another one.

Previous studies have demonstrated that red deer in the northeast of China tend to choose habitats with lower altitudes (800–1200 m) and slopes (<15°) [76]. The study area has a gentle slope, and the altitude ranges from 700 m to 1600 m. The large elevation fluctuation might increase the dispersal cost of red deer. During the sampling, it was found that the vegetation near the sampling sites did not change obviously, and *Quercus mongolica*, *Betula platyphylla*, *Ostryopsis davidiana*, and Poaceae could always be found around the sampling sites. Therefore, the habitat types and NDVI index did not show an apparent influence on the distribution and dispersal of red deer. The number of microsatellite loci, sample size, and analysis methods might limit the results of this study. More microsatellite loci and diversified analysis could provide more accurate and stable results on the genetic diversity of red deer. In future studies, we will apply more technical means to the genetic analysis of red deer while, at the same time, selecting more loci and larger sample sizes.

## 5. Conclusions

Understanding the current genetic structure and gene flow between the subpopulations is important to protect red deer in China. In this context, we investigated the genetic status of red deer living in the southern part of the Greater Khingan Mountains, China, to understand the effects of landscape environmental factors on gene flow. In this region, human activities have interfered with the movement and dispersal of red deer to some extent. In the long run, the red deer might have genetic risks. Human activities and traffic intensity should be supervised and controlled strictly, especially during the heat season (September to October). To strengthen the genetic diversity level and gene flow between different red deer subpopulations, it is necessary to establish ecological corridors to reduce dispersal resistance and promote gene exchange. In further research, a larger sample size, different molecular makers, and more complex analyses are necessary. This study helps us understand the genetic status and gives references for protecting and managing red deer resources in China.

## Figures and Tables

**Table 1:** The environment variables used in the MaxEnt model and their sources.

Environmental Variables		Year	Type of Variables	Source
Vegetation	NDVI	2016	Continuous variables	http://modis.gsfc.nasa.gov (accessed on 20 December 2022)
	Habitat types	2021	Categorical variables	The Third National land resource survey
Topography	Elevation	2009	Continuous variables	http://www.gscloud.cn (accessed on 20 December 2022)
	Slope		Continuous variables	http://www.gscloud.cn (accessed on 20 December 2022)
Water	Rivers	2015	Continuous variables	Euclidean distance layer between rivers and red deer sites was obtained and calculated by ArcGIS extraction
Disturbance	Settlements	2020	Continuous variables	Euclidean distance layer between settlements and red deer sites was obtained and calculated by ArcGIS extraction
	Roads	2020	Continuous variables	Euclidean distance layer between roads and red deer sites was obtained and calculated by ArcGIS extraction

**Table 2:** Information about the feces sample of red deer.

Sampling Site	R1	R2	R3	R4	R5	ALL
Sample size	29	57	55	61	29	231
Number of successful amplification sample size	27	53	54	54	24	212
Red deer samples	26	53	48	51	21	199
Successfully genotyping samples	26	53	44	51	19	193
Identified red deer individuals	22	45	39	47	19	172

**Table 3:** The amplification information for eight microsatellite primers.

Locus	*k*	*N*	*H*_o_	*H*_e_	*PIC*
ETH225	20	166	1.000	0.880	0.867
T501	7	172	0.657	0.707	0.657
T156	14	168	0.685	0.872	0.857
BM848	10	163	0.540	0.652	0.629
T530	10	166	0.783	0.783	0.756
T507	9	171	0.602	0.713	0.665
DM45	12	169	0.953	0.878	0.862
N	3	156	0.994	0.589	0.502

Note: *k*: number of alleles; *N*: number of samples; *H*_o_: observed heterozygosity; *H*_e_: expected heterozygosity; *PIC*: polymorphism information content.

**Table 4:** Genetic distances between the five geographic populations sampled.

Geographic Population	R1	R2	R3	R4	R5
R1					
R2	0.0041 *				
R3	0.0119 *	0.0139 *			
R4	0.0082 *	0.0223 *	0.0170 *		
R5	0.0111 *	0.0187 *	0.0128	0.0056	

Note: * indicated *p* < 0.01.

**Table 5:** Comparison of genetic diversity in the *Cervus* populations among different regions.

Species	Region	Microsatellite	
		*H*_o_	*H*_e_
*Cervus* *canadensis xanthopygus*	The southern part of the Greater Khingan Mountains, China	0.767	0.737
*Cervus canadensis xanthopygus* [28]	The southern part of the Greater Khingan Mountains, China	0.654	0.659
*Cervus elaphus alashanicus* [60]	Helan Mountains, Ningxia and Inner Mongolia, China	0.792	0.596
*Cervus elaphus wallichi* [27,62]	Sangri, Tibet, China	0.519	0.719
*Cervus elaphus yarkandensis* [63,64]	Tarim Basin, Xinjiang, China	0.083	0.378
*Cervus elaphus songaricus* [61]	Tianshan Mountains, Xinjiang, China	0.850	0.710
*Cervus elaphus scoticus* [65]	Scotland and England, Britain	0.447	0.801

## Data Availability

The raw sequences of the mitochondrial cytochrome *b* gene for this study can be found in GenBank from NCBI (submission ID: 2619872 OP373204-OP373385).

## Supplementary Materials

The following supporting information can be downloaded at: https://www.mdpi.com/article/10.3390/biology12040576/s1, Table S1: PCR amplification system of red deer for mtDNA and microsatellite; Table S2: Details of eight microsatellite loci used for the study; Figure S1: The relationship between dispersal resistance of red deer groups and environmental variables.
