# Supplementary material for: The Effect of Landscape Environmental Factors on Gene Flow of Red Deer (Cervus canadensis xanthopygus) in the Southern of the Greater Khingan Mountains, China
Source: Biology (Basel). 2023 Apr 10;12(4):576. doi: 10.3390/biology12040576 (PMC10135690; doi:10.3390/biology12040576)
Supplement: Supplementary file 1 [file biology-12-00576-s001.zip › Figure S1.pdf]

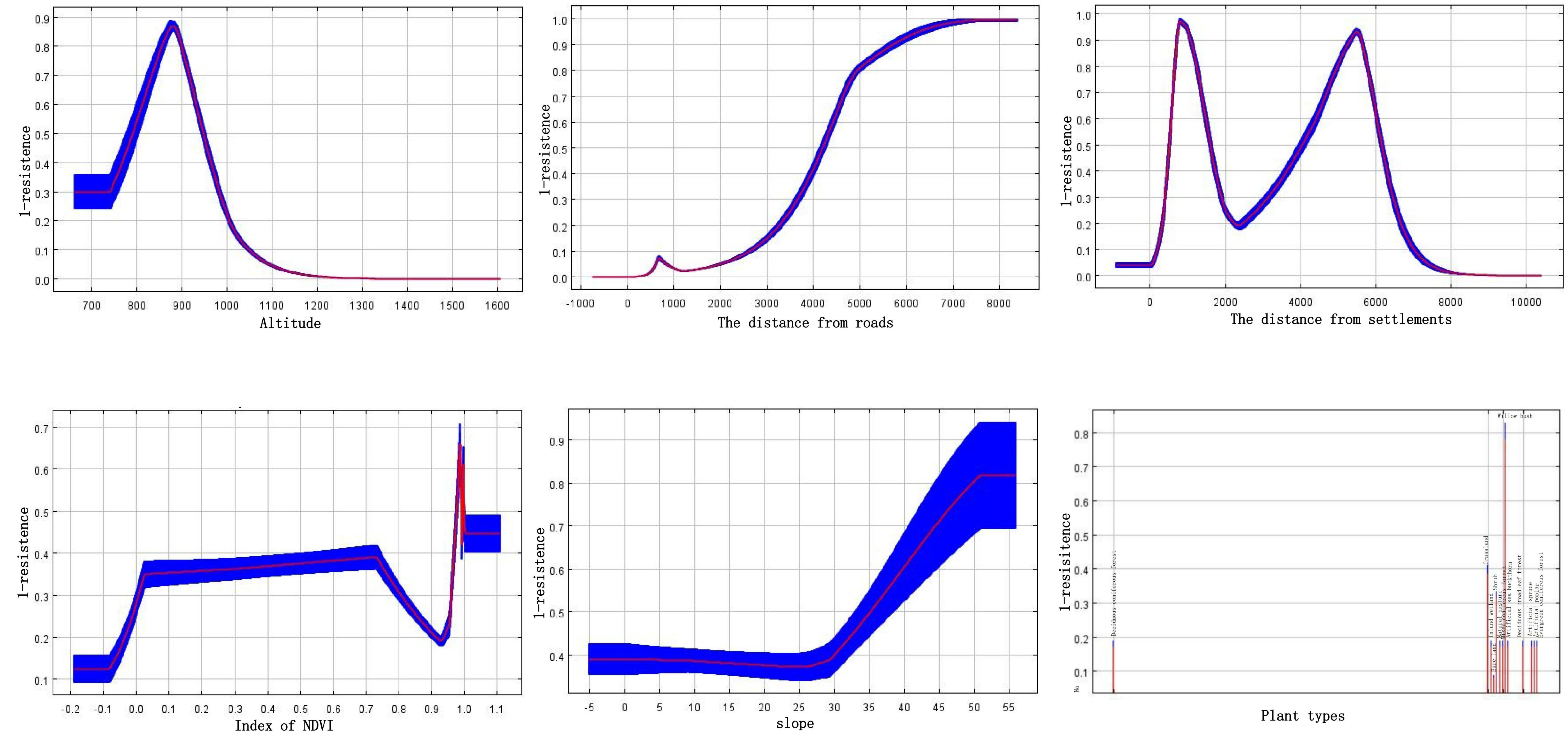

Figure S1: The relationship between dispersal resistance of red deer groups and environmental variables.
