# Supplementary material for: The Effect of Landscape Environmental Factors on Gene Flow of Red Deer (Cervus canadensis xanthopygus) in the Southern of the Greater Khingan Mountains, China
Source: Biology (Basel). 2023 Apr 10;12(4):576. doi: 10.3390/biology12040576 (PMC10135690; doi:10.3390/biology12040576)
Supplement: Supplementary file 1 [file biology-12-00576-s001.zip › Table S1.pdf]

Table S1. PCR amplification system of red deer for mtDNA and microsatellite

Reagent	Volume (μl)	
	Cyt *b*	Microsatellite
2×Rapid Taq Master Mix (China, Vazyme)	10.0	10.0
Forward Primer (10 μMol /L)	0.8	0.8
Reverse Primer (10 μMol /L)	0.8	0.8
DNA (25-50 ng/μL)	2.0	6.0
ddH^2O	6.4	2.4
Total	20.0	20.0
