# Supplementary material for: The Effect of Landscape Environmental Factors on Gene Flow of Red Deer (Cervus canadensis xanthopygus) in the Southern of the Greater Khingan Mountains, China
Source: Biology (Basel). 2023 Apr 10;12(4):576. doi: 10.3390/biology12040576 (PMC10135690; doi:10.3390/biology12040576)
Supplement: Supplementary file 1 [file biology-12-00576-s001.zip › Table S2.pdf]

**Table S2.** Details of 8 microsatellite loci used for the study

| Locus | Primer sequence (5'-3') | Allele Length (bp) | Repeat type | Annealing temperature (°C) |
|---|---|---|---|---|
| ETH225[1] | F:gatcaccttgccactatttcct<br>R:acatgacagccagctgctact | 133-185 | 2 | 56.0 |
| T501[2] | F:ctcctcattattaccctgtgaa<br>R:acatgctttgaccaagacc | 238-262 | 4 | 55.0 |
| T156[3] | F:tcttcctgacctgtgtcttg<br>R:gatgaatacccagtcttgtctg | 135-207 | 4 | 56.4 |
| BM848[4] | F:tggttggaaggaaaacttgg<br>R:cctctgctcctcaagacac | 350-366 | 2 | 54.5 |
| T530[4] | F:gtcctcacagcagctctatg<br>R:gcattctttagaactccaactg | 244-292 | 4 | 55.0 |
| DM45[3] | F:caccgtttcttacaatctca<br>R:aggggtcaggttctcagtttctac | 440-460 | 2 | 55.5 |
| N[2] | F:tccagagaagcaaccaatag<br>R:gtgtgccttaaacaacctgt | 282-290 | 4 | 56.0 |
| T507[3] | F:aggcagatgcttcaccatc<br>R:tgtggagcacctcacacat | 144-176 | 4 | 56.6 |

[1]*Tamra*,[2]*Rox*,[3]*Fam*,[4]*Hex*
